# Genetic Mechanisms Contribute to the Development of Heart Failure in Patients with Atrioventricular Block and Right Ventricular Apical Pacing

**DOI:** 10.1038/s41598-017-11211-2

**Published:** 2017-09-06

**Authors:** Nana Liu, Min Zheng, Shijie Li, Hui Bai, Zhouying Liu, Cui hong Hou, Shu Zhang, Jielin Pu

**Affiliations:** 1 State Key Laboratory of Cardiovascular Disease, Fuwai Hospital, National Center for Cardiovascular Diseases, Chinese Academy of Medical Sciences and Peking Union Medical College, Beijing, 100037 People’s Republic of China; 2 Department of cardiovascular diseases, Dongfang Hospital Affiliated to Tongji University, Shanghai, 200120 People’s Republic of China

## Abstract

Right ventricular apical (RVA) pacing can lead to progressive left ventricular dysfunction and heart failure (HF), even in patients with normal cardiac structure and function. Our study conducted candidate gene screening and lentivirus transfected neonatal rat cardiomyocytes (NRCMs) to explore the genetic and pathogenic mechanisms of RVA pacing induced cardiomyopathy in third degree atrioventricular block (III AVB) patients. We followed 887 III AVB patients with baseline normal cardiac function and RVA pacing. After a median follow-up of 2.5 years, 10 patients (four males, mean age 47.6 ± 10.0 years) were diagnosed with RVA pacing induced HF with left ventricular ejection fraction (LVEF) reducing dramatically to 37.8 ± 7.1% (P  < 0.05). Candidate genes sequencing found cardiomyopathy associated genetic variations in all ten HF patients and six SCN5A variations in 6 of 20 control patients. Transfected NRCMs of Lamin A/C mutations (R216C and L379F) disrupted Lamin A/C location on nucleus membrane and finally resulted in increased apoptotic rate after serum starvation. In conclusion, cardiomyopathy associated genetic variations play an essential role in occurrence of newly onset HF in the III AVB patients with RVA pacing. RVA pacing, serving as extra stimulator, might accelerate the deterioration of cardiac structure and function.

## Introduction

As the only effective therapy for bradycardia, millions of permanent cardiac pacemakers are implanted worldwide every year. However, as previous studies have verified, right ventricular apical (RVA) pacing can cause electrical and mechanical dyssynchrony and eventually induce adverse effects on myocardial metabolism and perfusion, remodeling, hemodynamics and mechanical function^1, 2^. Several large randomized controlled trials (RCTs) have confirmed the correlation between long-term RVA pacing and deterioration of heart structure and function, especially in patients with high RVA pacing burden and impaired baseline left ventricular ejection fraction (LVEF)^3–6^. Usually, symptomatic patients with third degree atrioventricular block (III AVB) need high percentage of RVA pacing and are more liable to develop HF. Nowadays, based on the studies on AVB patients with normal or nearly normal baseline LVEF, the incidence of newly-onset HF induced by RVA pacing varies a lot, from 3.2% to 26%; and similar variation was found in the timing of onset, from 1 month to more than one decade, probably due to different recipient populations and follow-up durations^7–14^. There are several risk factors such as old age^9, 14^, male gender^11^, coronary artery disease^9^, wider native QRS duration (QRSd)^11, 13^ or wider paced QRSd^9, 12^ for RVA pacing cardiomyopathy.

The pathogenesis of HF involves a complex interaction between genetic and environmental factors. Genetic factors may influence the susceptibility to the underlying etiology of HF, the rapidity of disease progression, or the response to pharmacologic therapy^15^. Moreover, HF can arise in the setting of a primary cardiomyopathy, most commonly, dilated cardiomyopathy (DCM); 30–50% of DCM cases are familial aggregation and caused by genetic mutations^16^, among them Lamin A/C and TTN are the most common mutations detected in DCM cohort. Clinical studies have shown that Lamin A/C*-*DCM is characterized by early-onset atrial fibrillation and conduction system disease, and subsequently, progression to HF and sudden cardiac death (SCD)^17–19^. Thus, we hypothesize that genetic mechanism plays an important role in the development of newly-onset HF in certain III AVB patients and long-term RVA pacing may accelerate the deterioration of cardiac structure and function. To evaluate this hypothesis, we investigated the genetic background of III AVB patients with RVA pacing induced cardiomyopathy and explored the pathogenic mechanism with transfected neonatal rat cardiomyocytes (NRCMs).

## Results

### Clinical characteristics of the study population

877 patients were enrolled in our study. Before pacemaker implantation, all patients were diagnosed with third degree atrioventricular block (III AVB) for more than 3 months. Clinical characteristics of the study population are summarized in Table 1. The mean age was 57.4 ± 18.4 years, 465 (52.9%) were male. 408 (46.4%) patients were diagnosed with hypertension, 93 (10.6%) with hypercholesterol, 94 (10.7%) with Diabetes mellitus, 51 (5.8%) with coronary heart disease and 104 (11.8%) had a history of AF. Echocardiography showed the baseline LVEF (%) and LVEDD (mm) at the time of pacemaker implantation were 65.3 ± 7.9 and 50.0 ± 7.8. The baseline heart rate was 42 ± 12 bpm. There are no clear etiologies of III AVB in 693 (78.8%) cases, and the other 186 patients exhibited potential causes that may lead to III AVB. It was clear that cardiac surgery (64, 7.3%) was the most common cause. Among all the patients, 683 patients (75.7%) were designed to DDD pacing mode and 194 patients (22.1%) were with VVI mode.

**Table 1:** Baseline clinical characteristics of the study population.

Basic Characteristics	n = **879**
Age (years)	57.4 ± 18.4
Male, n (%)	465 (52.9)
Hypertension	408 (46.4)
Hypercholesterol	93 (10.6)
Diabetes Mellitus	94 (10.7)
Coronary artery disease	51 (5.8)
History of AF, n (%)	104 (11.8)
Etiologies	
None, n (%)	693 (78.8)
Cardiac surgery	64 (7.3)
Cardiomyopathies, n (%)	26 (3.0)
HCM	20 (2.3)
RCM	5 (0.5)
LVNC	1 (0.1)
Rheumatic heart disease	25 (2.8)
Myocardial infarction	24 (2.7)
Viral myocarditis	20 (2.3)
RFA	15 (1.7)
Congenital third degree AVB	12 (1.4)
Baseline LVEF (%)	64.3 ± 7.1
Baseline LVEDD (mm)	49.9 ± 6.8
Baseline HR (bpm)	42 ± 12
Pacing mode, n (%)	
DDD	683 (75.7)
VDD	20 (2.2)
VVI	194 (22.1)
Mean percentage of ventricular pacing, (%)	99.0 ± 2.3

AF, Atrial Fibrillation; AVB, Atrioventricular Block, HCM, Hypertrophic Cardiomyopathy, HR, Heart Rate; LVNC, Left Ventricular Non-compaction Cardiomyopathy; RCM, Restricted Cardiomyopathy; RFA, Radiofrequency Ablation.

## Follow up

### The whole population

After a median follow-up of 2.5 years, the average pacing percentage of the study population is 99.0%. Echocardiography showed the average LVEF of the whole population decreased significantly from 65.3 ± 7.9 to 60.7 ± 8.5 (p <0.001) and there was no significant change in LVEDD (mm, 50.0 ± 7.8 VS. 49.9 ± 6.8, p = 0.748). In total, 31 (3.53%) were readmitted to Fuwai hospital because of refractory HF. The echocardiography of 31 patients showed significant decrease in LVEF (%, from 62.0 ± 5.6 to 35.9 ± 7.2, p < 0.001) and increase in LVEDD (mm, from 54.0 ± 6.9 to 57.7 ± 7.6, p = 0.014). Among the 31 newly-onset HF patients, 18 cases were found with MI, 2 with HCM and 1 with AF and rapid ventricular pacing according to the medical records. Ten patients with no alternative causes for HF were diagnosed as RVA pacing induced cardiomyopathy.

### RVP associated HF

Within 20.0 ± 10.2 months, all 10 cases (4 males, mean age of 47.6 ± 10.0 years) developed severe HF and were diagnosed with RVP induced HF. Compared with the baseline echocardiography, LVEF (%) reduced significantly from 60.1 ± 5.6 to 37.8 ± 7.1 (P <0.05); meanwhile, LVEDD (mm) increased remarkably from 54.2 ± 4.9 to 61.0 ± 4.7 (P <0.05). Nine of them were diagnosed as NYHA class II with one assigned to NYHA class III. Genetic testing revealed 15 cardiomyopathy associated genetic variations (10 pathogenic and 6 VUS) found in the 10 HF patients. Three patients with Lamin A/C variations (Lamin A/C p. Leu379Val, c.1157 + 1 G > T and c.1157 + 1 G > T) and one with MYBPC3 p. Gly507Arg mutations were found to have a family history of SCD. The Clinical characteristics and genetic results of HF patients are displayed in Table 2.

**Table 2:** Clinical characteristics and genetic testing results of the HF patients.

Patients	Age/Gender	Family History of SCD	Arrhythmias	Risk Factors	Pacing Mode	Baseline Echocardiography		Duration of HF Onset (months)	Follow-up Echocardiography		Genetic Testing Results	Mutation Prediction
						LVEF (%)	LVEDD (mm)		LVEF (%)	LVEDD (mm)		
1	39/M	Yes	III AVB, AF	HT	VVI	70	57	28	39	59	*LMNA* p. Leu379Val	Pathogenic
2	41/M	Yes	III AVB, AF, PVC, VT	None	VVI	63	56	13	31	65	*LMNA* c.1157 + 1 G > T	Pathogenic
3	57/M	None	III AVB	None	DDD	64	47	12	45.5	58	*TTN* p. Glu5365Asp; *TTN* p.Arg3067His	Pathogenic; VUS
4	37/F	None	III AVB	None	DDD	53	62	15	42.5	70	DSP c.1140 + 6 T > C	VUS
5	54/F	None	IIIAVB, PVC, VT, VF	None	DDD	62	55	30	25	64	*CACNA1C* c.372-9 C > G; *CACNA1C* c.4074 + 6 C > T	VUS; VUS
6	64/F	Yes	III AVB, AF	HT	DDD	52	52	25	35.6	54	*LMNA* p. Arg216Cys	Pathogenic
7	39/F	None	III AVB	None	DDD	60	57	6	39	59	*DSG2* p. Leu563Arg; *AKAP9* c.9358 + 10 A > G	VUS; VUS
8	46/M	None	III AVB, CLBBB	None	DDD	60	55	28	45	59	*TTR* p. Ser43Asn; *DSG2* p. Phe531Cys; *CSRP3* p. Gly72Arg	Pathogenic; Pathogenic; Pathogenic
9	60/F	None	III AVB	HT	DDD	63	47	24	28	61	*MYH7* p. Glu1902Gln; *TTN* p. Arg8985Cys	Pathogenic Pathogenic
10	39/M	Yes	III AVB	None	DDD	54	54	8	42	58	*MYBPC3* p. Gly507Arg	Pathogenic

AVB, Atrioventricular Block; AF, Atrial Fibrillation; CLBBB, Complete Left Bundle Branch Block; LVEF, Left Ventricular Ejection Fraction; LVEDD, Left Ventricular End Diastolic Dimension; PVC, Premature Ventricular Contraction; HT, Hypertention; SCD, Sudden Cardiac Death; VT, Ventricular Tachycardia; VUS, Variation of Unknown Significance.

During the follow-up period, eight patients with RVP induced HF remained viable, while one (patient 2) died of SCD after contracting an upper respiratory infection and one (patient 10) died of chronic refractory HF. Twelve months after pacemaker implantation, patient 2 visited our emergency department because of palpitation. ECG showed sustained ventricular tachycardia with HR 148 bpm and BP 90/75 mmHg. Despite the use of anti-HF drugs and amiodarone, he suffered SCD 16 months later. In spite of taking anti-HF drugs, patient 5 suffered serious symptoms of HF and finally updated to CRT after 2 years’ RVA pacing. Her LVEF were 39%, 29%, 29%, 39% and 37% at 12, 26, 36, 42 and 72 months after CRT implantation, respectively.

### Control group

After matching with age, gender and time of enrollment, a total of 20 patients with III AVB and no history of HF were enrolled as the control group (2 controls for each HF patients). At the time of pacemaker implantation, according to echocardiography results, the mean LVEF of the control group is significantly higher than the HF patients (%, controls vs. HF, 66.9 ± 7.9 vs. 60.1 ± 5.6, P = 0.019), however mean LVEDD (controls vs. HF: 50.6 ± 5.6 mm vs. 54.2 ± 4.6 mm, P = 0.096) did not show any significant difference. After nearly 30 months of RVA pacing, the control group suffered nearly 5% decrease in LVEF (from 66.9 ± 7.9% to 61.6 ± 5.7%, P < 0.01), but no one exhibited HF symptoms or LVEF below 50%. Meanwhile LVEDD did not significantly alter (baseline vs. follow-up, 50.7 ± 5.6 mm vs. 50.4 ± 4.7 mm; P = 0.807).

We did not detect any genetic mutations in 14 of the 20 controls. Six different loci variations of SCN5A were found in six patients. Of them, SCN5A p. Arg1193Gln was found uniquely or jointly with other SCN5A variations (p. Arg513Cys and c.2023 + 10 C > T) in four patients. Clinical characteristics and genetic testing results of control group were listed in Table 3.

**Table 3:** Clinical characteristics and genetic testing results of the control group.

Patients	Age/Gender	Arrhythmias	Risk Factors	Pacing Mode	Baseline Echocardiography		Follow-up Echocardiography		Genetic Testing Results
					LVEF (%)	LVEDD (mm)	LVEF (%)	LVEDD (mm)	
1	57/M	III AVB, AF	None	VVI	65	55	50	55	SCN5A p. Lys590 Gln
2	41/M	III AVB, AF	None	VVI	52	58	50	49	None
3	37/M	III AVB	None	DDD	63	44	61	50	None
4	64/F	III AVB	HT	DDD	70	55	60	60	None
5	64/F	III AVB	HT	DDD	66	58	59	55	SCN5A p. Arg1193 Gln
6	39/M	III AVB	None	DDD	70	50	60	57	SCN5A p. Arg1193Gln
7	34/F	III AVB	None	DDD	67	43	63	40	None
8	39/F	III AVB	None	DDD	76	60	65	53	None
9	34/F	III AVB	None	DDD	50	50	60	47	SCN5A p. Arg1193Gln; SCN5A c.2023 + 10 C > T
10	64/F	III AVB	None	DDD	65	55	67	53	None
11	64/F	III AVB	HT	DDD	68	42	71	50	SCN5A p. Arg1193Gln; SCN5A p. Arg513Cys
12	39/M	III AVB	None	DDD	77	59	70	53	SCN5A p. Ala1126Val
13	41/M	III AVB	None	DDD	73	49	66	51	None
14	57/M	III AVB, AF	DM	VVI	64	52	60	54	None
15	39/M	III AVB	None	DDD	79	51	60	49	None
16	39/F	III AVB	None	DDD	65	48	53	46	None
17	60/F	III AVB	HT	DDD	63	47	61	48	None
18	58/F	III AVB	None	DDD	67	45	65	45	None
19	40/M	III AVB, PVC	None	DDD	63	47	64	47	None
20	42/M	III AVB	None	DDD	68	45	66	46	None

AVB, Atrioventricular Block; AF, Atrial Fibrillation; LVEF, Left Ventricular Ejection Fraction; LVEDD, Left Ventricular End Diastolic Dimension; PVC, Premature Ventricular Contraction; HT, Hypertention.

### Characterizations of Lamin A/C mutations in NRCMs

**Figure 1:**
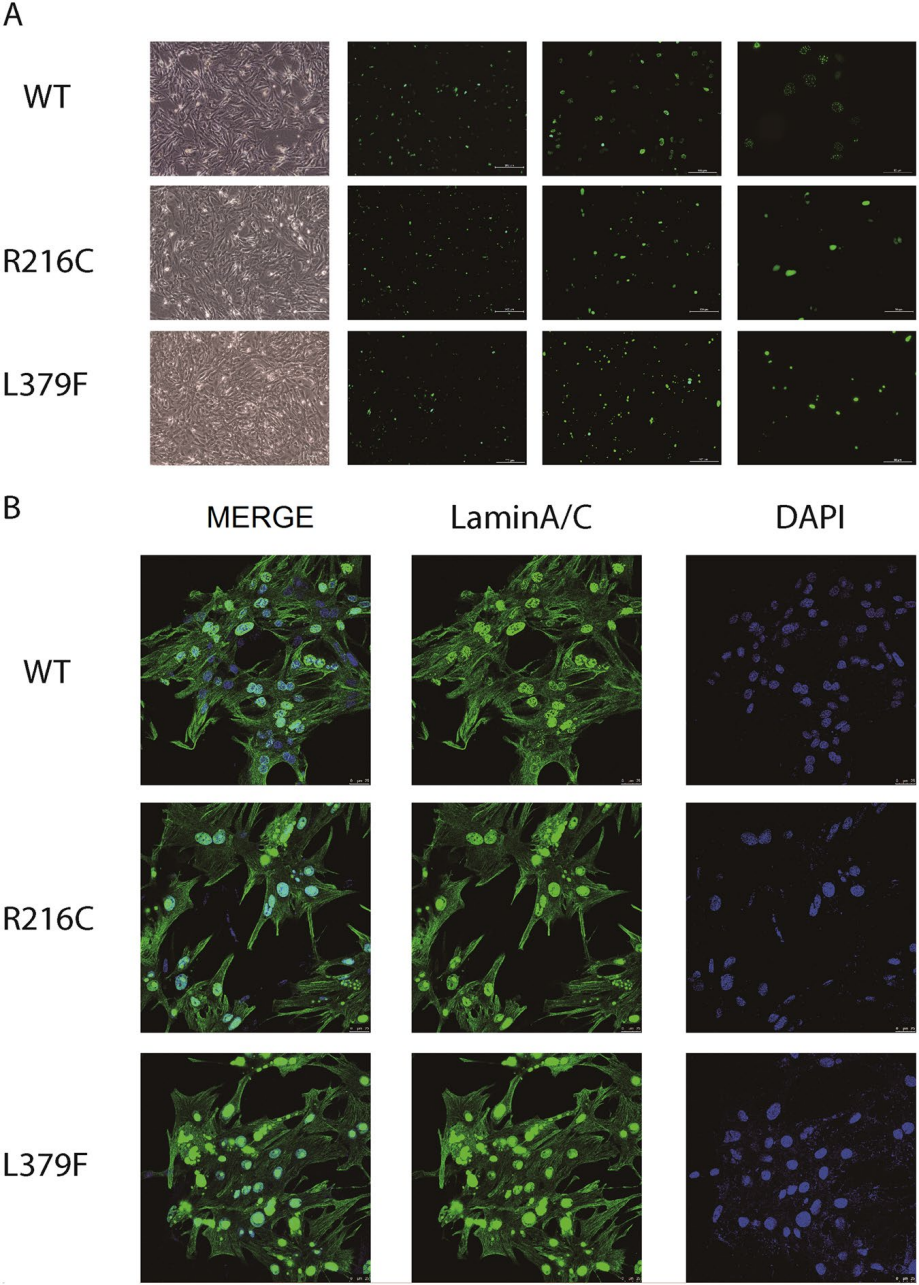
Nuclear location of Lamin A/C by fluorescence microscope. (**A**) The WT NRCMs showed regular rounded nucleus and lamin A/C demonstrated as small aggregates homogenously distributed in the nucleus envelope; (**B**) Lamin A/C mutations (R216C and L379F) showed nucleus with irregular shape (→), lamin A/C appeared profoundly impaired, clearly expressed in highlighted aggregates of different sizes, not uniformly distributed along the nucleus envelope.

To functionally characterize the newly identified Lamin A/C mutations, Neonatal rat cardiomyocytes (NRCMs) were transfected with lentviruses of Lamin A/C WT and two mutations (R216C and L379 F). Fluorescence microscope was used to determine the nuclear localization. As expected, NRCMs expressing Lamin A/C WT showed regular rounded nucleus and Lamin A/C proteins demonstrated as small aggregates homogenously distributed in the nucleus; In contrast, Lamin A/C mutations had irregular nucleus, the localization of Lamin A/C proteins appeared profoundly impaired, clearly expressed in highlighted aggregates of different sizes, not uniformly distributed along the nuclear envelope (Fig. 1).

**Figure 2:**
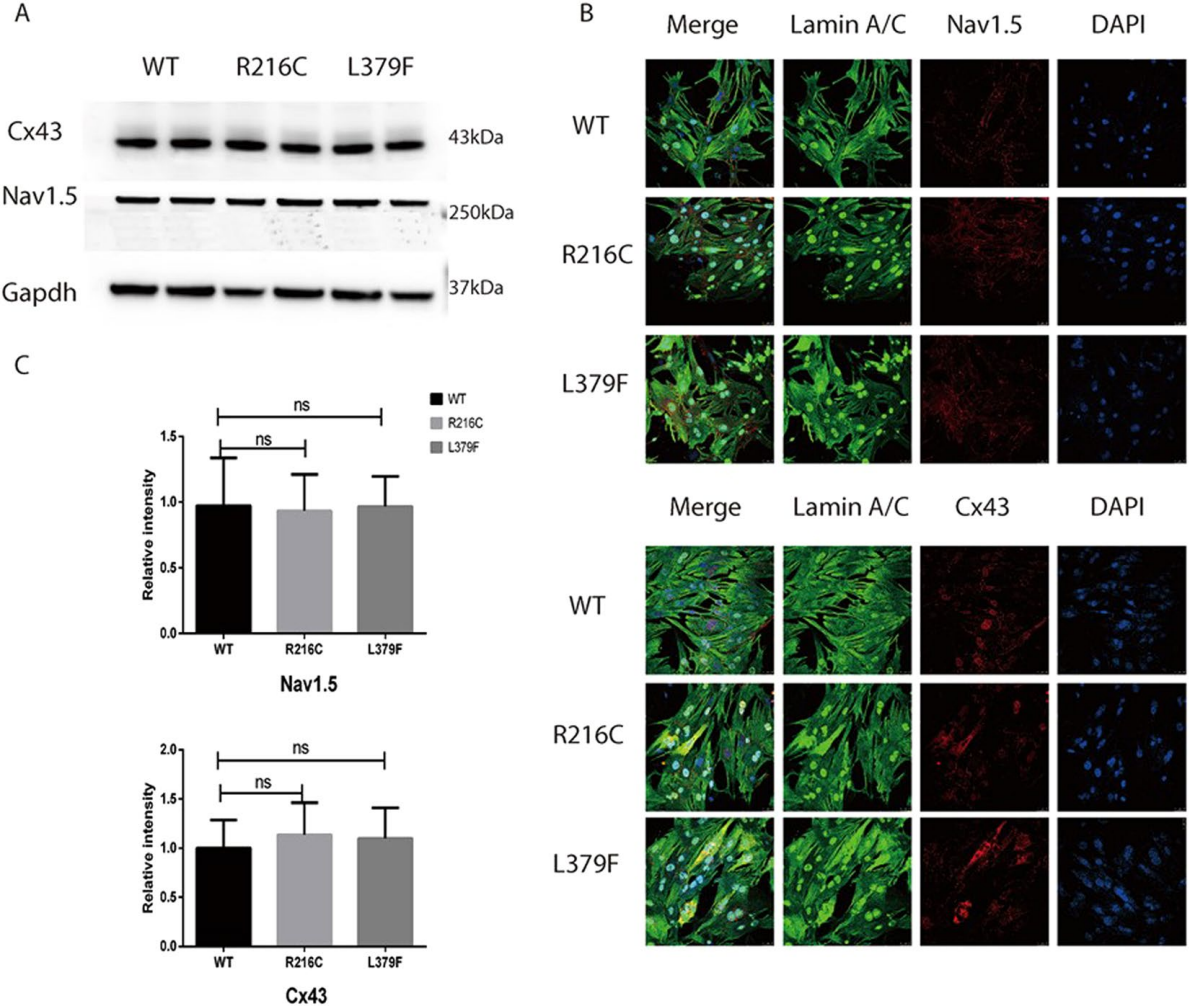
No significant differences on the location and expression of Nav1.5 or Cx43 between Lamin A/C WT and mutations. (**A**) Western blot showed equal expression level of Nav1.5 and Cx43; (**B**,**C**) Confocal imaging did not reveal significant difference on the expression either in nucleic membrane or in cytomembrane. ns, not significant.

As patients with Lamin A/C mutations (R216C and L379F) developed III AVB before HF, we hypothesized whether defects in sodium channels and/or Connexin 43(Cx43) accounted for the evolution of cardiac electrophysiological dysfunction. However, Western blot did not reveal any significant differences in the expression levels of Nav 1.5 and Cx43. On the other hand, compared with Lamin A/C WT, there were no significant differences in the location and expression of Cx43 or Nav1.5 in the nucleic membrane and cytomembrane of NRCMs of Lamin A/C mutations (Fig. 2).

### NRCMs apoptosis

**Figure 3:**
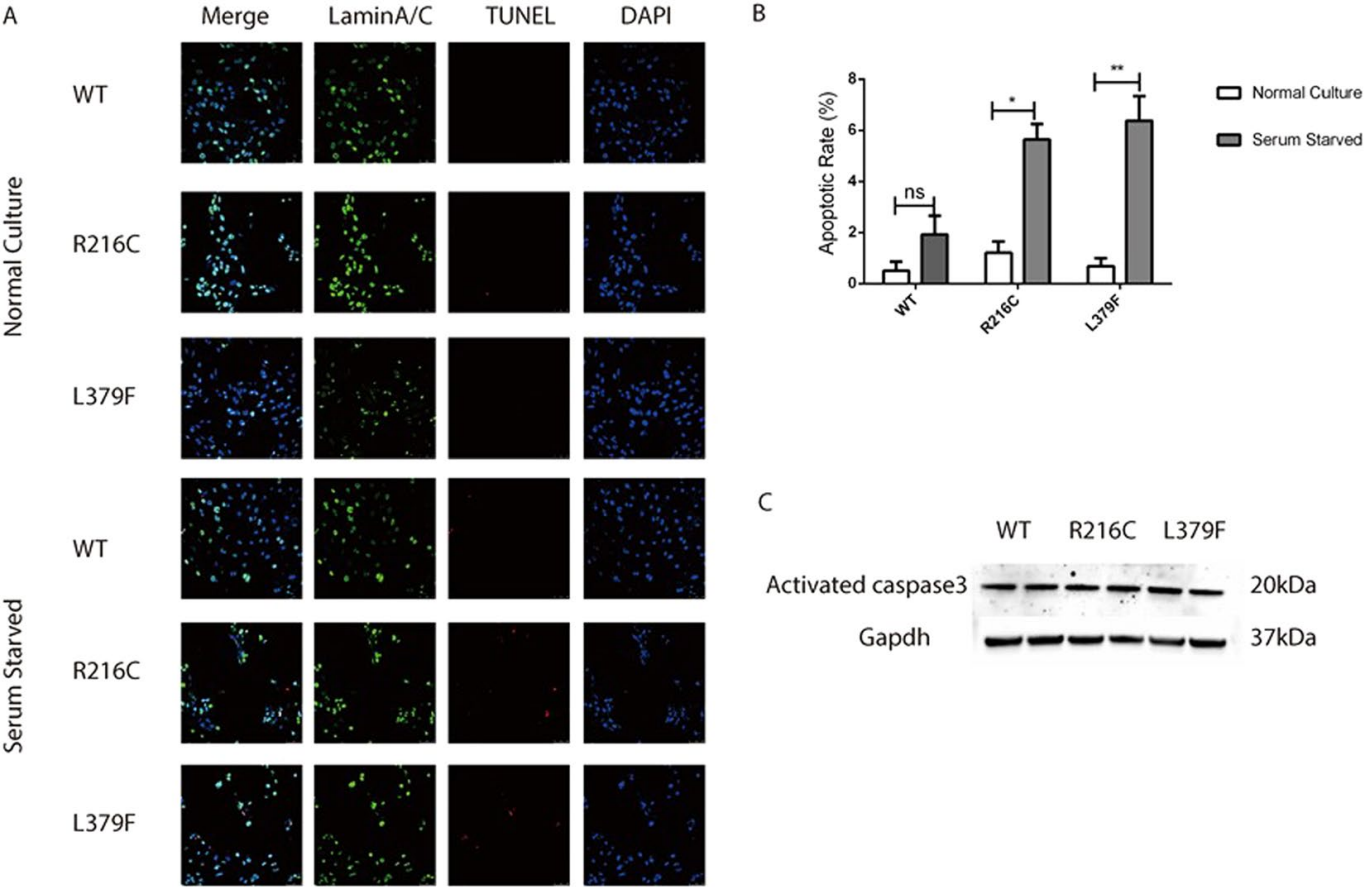
Apoptotic rate assessed by TUNEL and Western blot detection of activated caspase 3 in normally cultured and serum starved Lamin A/C NRCMs. (**A**) Confocal imaging of TUNEL assay showing normally cultured Lamin WT, R216C and L379 had similar apoptotic rate. HoweverLamin A/C R216C and L379F with serum starvation had significantly increased apoptotic rate. (**B**) Quantification of apoptotic rate was valued. (**C**) After serum starvation, there is no significant difference in the expression level of activated caspase 3.*p-value < 0.05; ns, not significant.

We hypothesized that progressive demise cardiomyocytes were responsible for the occurrence of AVB and HF. To further verify this hypothesis, we studied the apoptosis of normally cultured NRCMs and serum starved NRCMs. The serum starved NRCMs were cultured normally with 5% Fetal Bovine serum (FBS) within the first 72 hours and then cultured without FBS for 24 hours. 96 hours after lentiviruses transfection, cells specimens were fixed and examined for apoptosis by deoxynucleotidyl transferase-mediated dUTP nick end labeling (TUNEL)., Compared with the normally cultured NRCMs, confocal imaging results showed there was no significant changes in the apoptotic rate in serum starved Lamin A/C WT cells (0.52% ± 0.33% vs. 1.03% ± 0.73%, n = 6, p = 0.109) Nevertheless, statistical increase in TUNEL positive cells were found in both Lamin A/C R216C cells (1.21% ± 0.44% vs. 6.64% ± 0.60%, n = 6, p < 0.05) and Lamin A/C L379F cells (0.68% ± 0.31% vs. 6.37% ± 0.97%, n = 6, < 0.05) after serum starvation (Fig. 3A,B). On the other hand, both Lamin A/C WT and mutations have similar apoptotic percentages under normal culture condition. However, there was no significant discrepancy in the expression level of activated caspase 3 under two different culture conditions (Fig. 3C), probably due to the relatively low percentage of apoptotic cells.

## Discussions

Our results suggested that for some III AVB patients who developed RVA pacing induced HF, the cardiomyopathy associated genetic mutations account for conduction abnormality as well as ventricular enlargement and cardiac dysfunction. RVA pacing acting as extra stimulator may accelerate the process of deterioration of cardiac dysfunction. NRCMs carried Lamin A/C mutations (R216C and L379F) showed disrupted Lamin A/C nuclear location and increased apoptotic rate under environmental stress. Genetic testing contributes to the detection of patients with high risk of HF as well as exploration of the pathogenic mechanism.

Currently, there are still some conflicting results about the effects of RVA pacing in patients without heart failure. It was found that high percentage of RVA pacing was associated with HF hospitalization in some large RCT of PM^5, 20–22^. However, some trials showed HF had nothing to do with RVA pacing^23^. Other risks such as older age, CAD and a wider paced QRS duration maybe associated with HF^9, 24^. Importantly, it took a long term, usually more than three years, to manifest LV dysfunction in patients with baseline normal LVEF^7, 9, 25, 26^. Furthermore, it has been shown that pacing location and sequence of cardiac electrical conduction have little effect on patients with normal baseline LVEF in the Protect-Pace study^27^ and in a large Japanese study^28^. Ruling out the risks of HF referred above,our study discovered that there were still a small amount of III AVB subjects of younger age (mean age, 47.1 ± 10.0 years) who developed new-onset HF within 3 years after PM implantation. This finding inspired us to explore other pathogenic mechanisms in the HF cases.

The major findings of this study revealed that for those who developed HF after RVA pacing, cardiomyopathy associated genetic mutations such as Lamin A/C mutations are the genetic background of conduction diseases and HF; RVA pacing could just be a coincidence, or a contributor. As the HF patients were diagnosed with AVB for more than three months, and HF occurred just after a short period of RVA pacing, it suggests pacing is the main inducement for HF. Cardiomyopathy associated genetic mutations play an essential role in the development of arrhythmias and finally progression to cardiac dysfunction. Wolf *et al*. showed that Lamin A/C haploinsufficiency led to early-onset programmed cell death of conducting cardiomyocytes such as atrioventricular nodal myocytes, and resulted in late-onset DCM and HF^29^. Therefore, electrically active cardiomyocytes might be more likely to be destroyed, which led to heart block. According to two large studies about the genetic background of DCM^16, 30^, there is a high percentage of overlap in the mutations causing DCM, HCM, ARVC and channelopathy. Remarkably, six cases in HF group carried compound or combined variations associated with DCM, HCM, ARVC, or channelopathy and the overlapped phenotypes of AVB and HF could be explained by at least part of the above findings. Mutations associated with inherited arrhythmia and cardiomyopathy may result in both a destroyed conduction system and fragile cardiac walls, once PM is implanted, the fragile heart can be destroyed with increased burden of heartbeat rate.

We hypothesized that long-term RVA pacing could accelerate the deterioration of cardiac structure and function in cardiomyopathy mutation carriers. Multiple experimental studies using *in-vivo* pacing model or adult rat ventricular cardiomyocytes have demonstrated that rapid electrical pacing could induce increased mitochondrial activity and myocardial activation of Akt, JNK, Erk or p38 MAPK as well as abnormal Ca^2+^ homeostasis which correlated with the rise in cardiomyocytes apoptosis^11, 31–34^. Furthermore, Siu *et al*. found that Lamin A/C‐mutated iPSC-CMs showed markedly increased percentage of nuclear senescence and cellular apoptosis under field electrical stimulation^35^. In our study, Lamin A/C mutations (R216C and L379F) could significantly change the location of Lamin A/C proteins and affect nuclear mechanical stability. In addition, as the apoptotic rate did not show any difference in the normally cultured Lamin A/C WT and mutated NRCMs, only after serum starvation the percentage of apoptosis increased, which on the other hand means that mutated cells are more susceptible to the adverse effect of environmental stress. Once the HF cascade was activated, multiple factors such as electrical stimulators can accelerate the causal pathways. Furthermore, pharmacological blockade of apoptotic pathways of ERK1/2 or MEK 1/2 pathway, such as U0126 and selumetinib (AZD6244), significantly attenuated the pro‐apoptotic effects of field electric stimulation on the mutated Lamin A/C iPSC‐CMs^35^ and Lamin A/C -DCM mouse models^36–38^.

Four control patients carried the same SCN5A mutation p. Arg1193Gln. This mutation was initially found in a Japanese patient who survived sudden unexplained nocturnal death. Patch clamp recording showed this mutation could shift the balance of current towards *I*to by accelerating the decay of *I*_Na_^39^. In addition, other studies in Han population found that this mutation was both a risk factor for LQTS^40–42^ and cardiac conduction defects^42, 43^. Without any signs of QTc elongation, our study adds more evidence of SCN5A p. Arg1139Gln being a genetic factor of cardiac conduction defects.

There were several risk factors discovered in the RVA induced HF patients, including family history of HF or SCD, younger age, combined brady/tachyarrthymias, lower baseline LVEF and/or ventricular enlargement before PM implantation, rapid decrease of cardiac function after PM implantation, negative reaction to anti-HF drugs. Given the high proportion of genetic variants detected in the HF cohort, that genetic testing can be considered if it will help in counseling first-degree relatives. As several studies have shown that CRT preserves LVEF, minimizes LV dyssynchrony and prevents adverse remodeling in patients with normal LVEF^8, 44^. For those who carry pathogenic genetic mutations, maybe it is more sensible and effective to get biventricular pacing instead of right ventricular pacing at the first time of device implantation. Block-HF study has shown promising results of CRT on patients with third degree atrioventricular block and left ventricular systolic dysfunction^27^. However in our study, only one HF patient updated to CRT treatment. In the future, we can explore the application prospect of CRT in those with RVA pacing induced HF patients.

### Study Limitations

This study has several limitations. Firstly, this was a retrospective study, and thus it cannot be excluded that patients with clinical heart failure obtained echocardiographic assessment more frequently and these patients were those with impending or subclinical laminopathy. Furthermore, our results came from a cohort from a single center in China, and did not account for genetic heterogeneity that is likely to be relevant when applied to other patient populations. Secondly, there were a limited number of controls underwent sequencing of candidate CMP genes and we failed to undergo cosegregation analysis of the SCN5A mutations identified in the control cohort. We evaluated their SNP frequencies and three databases (PolyPhen-2, SIFT and Mutation_Taster) were used to evaluate the function of SCN5A variations. Two mutations SCN5A p. Arg1139Gln^39–43^ and p. Ala1126Val^45^ have been shown to be pathogenic. However, other mutations (p. Lys590Gln, c.2023 + 10 C > T and p. Arg1126Cys) were VUS and pedigree validation was not available. In future study, we will perform more comprehensive genetic testing in a larger III AVB population to find more genetic evidence. Thirdly, specific pathogenic mechanisms of RVA pacing on the development of HF remains unclear and needs to be addressed by introduction of electrical filed stimulation in transgenic animal models to explain the effect of RVA pacing on mutated cardiomyocytes. Fourthly, the hospitalization rate of newly-onset HF is 1.1% in our study, similar to it of Protect-Pace study^27^, but lower than the HF rate of published randomized clinical trials^8–25^. There are two reasons: for one, HF symptoms and signs are not specific for the diagnosis of HF^46^. Some patients with mild HF symptoms are unaware of their pathogenic condition and some may just visit their local hospitals. Another reason, the average age in our study is 57.4 years old, which is much lower than those in RCTs (over 70 years old). Younger patients have less underlying diseases and are less likely to develop HF. Besides, most HF patients receive treatment in the outpatient clinic in our hospital, only those with severe HF symptoms and failed to respond to anti-HF drug therapy were hospitalized.

## Conclusions

In conclusion, cardiomyopathy associated genetic mutations play an essential role on occurrence of III AVB and HF after RVA pacing in a small proportion of patients. RVA pacing, acting as an extra stimulator, can accelerate the deterioration of cardiac structure and function. Lamin A/C mutations (R216C and L379F) can disrupt nuclear Lamin A/C location and lead to apoptosis of NRCMs after serum starvation. Genetic testing serves as an effective way to identify patients with high risk of HF and pathogenic mechanism of RVA induced cardiomyopathy.

## Methods

### Study Population

We conducted a retrospective study of AVB patients, who have underwent pacemaker implantation at Fuwai Hospital from January 1987 to December 2013. Enrollment criteria included: III AVB; Baseline left ventricular ejection fraction (LVEF) ≥ 50%; Single-chamber /dual-chamber pacemaker was implanted with RV lead positioned at RV apical; Repeat echocardiography was available after pacemaker implantation. To assess left ventricular volume and ejection fraction, we used real-time three-dimensional echocardiography (with the iE33 system, Philips) in 86% of the patients, whereas the biplane Simpson’s method was used in the other 14%. The echocardiographic images were stored and sent to the core laboratory for analysis by echocardiographic specialists in a blinded fashion. All patients provided written informed consent. Design of this study and informed consent were approved by our local ethics committee (Ethics Committee of Fuwai Hospital affiliated to Peking Union Medical College), and were carried out in accordance with the principles of the Declaration of Helsinki.

### RVP associated HF

We reviewed patients’ medical records and echocardiography after pacemaker implantation. HF was defined as more than 10% decrease in LVEF with resultant LVEF <50%. Patients who developed HF and identified with an alternative causes of HF such as myocardial infarction, myocardial ischemia on stress testing, severe valvular heart disease, atrial arrhythmias with rapid ventricular response, uncontrolled hypertension and frequent (å 25%) premature ventricular contraction, were excluded. Uncontrolled hypertension was defined as ≥ 50% of measured blood pressure exceeding 160/100 mmHg according to Frammingham Heart Study^47^. In total, 10 patients with RVA pacing associated HF were included in our study. In addition, we included 20 III AVB patients matched for age and gender as the control group.

### Mutational analysis

Genomic DNA was extracted from peripheral blood leukocytes using a TIANamp Blood DNA isolation kit (Tiangen, Beijing, China) according to the manufacturer’s instructions. The coding and flanking regions of 61 genes associated with inherited arrhythmia and cardiomyopathy were amplified by a custom designed library (Agilent Technologies, Santa Clara, CA, USA) and subsequently sequenced on Genome Analyzer IIx (Illumina Inc, CA, USA). The mutation was confirmed by Sanger’s method. Whenever the mutation or single nucleotide polymorphism was found in the proband or her family members, it would be confirmed in 500 unrelated healthy Chinese individuals. A variant was considered as a common polymorphism according to human reference genome (http://www.1000genomes.org/) or Exome Variant Server (http://evs.gs.washington.edu/EVS/).The remaining variants were confirmed by Sanger capillary sequencing and confirmed in 500 unrelated healthy Chinese individuals. A sequence variant was considered to be pathologic if it was absent in 500 ethnically matched healthy controls, and with one of the following criteria: (1) a deletion mutation (2) a missense mutation indicated a damaging effect by anyone of PolyPhen-2 (http://genetics.bwh.harvard.edu/pph2/), SIFT (http://sift.bii.a-star.edu.sg/www/SIFT_ BLink_submit.html/) and Mutation_Taster (http://www.mutationtaster.org/). A variant was regarded as Variation of Unknown Significance (VUS) if there were no associated reports and the bioinformatics software produced discrepant results.

### Primary culture of neonatal rat cardiomyocytes (NRCMs)

Neonatal rat cardiomyocytes were isolated and cultured using the method previously described^48, 49^. The Institutional Animal Care and Use Committee of Fuwai Hospital approved all experimental procedures. All methods were performed in accordance with the relevant guidelines and regulations. Neonatal hearts were placed in an ice-cold Hanks balanced salt solution (HBSS). The apex of the ventricular from the lower 1/3 of the heart was separated and cut into smaller pieces. The pieces were incubated in 1 ml of digestion buffer (0.25 mg Liberase^™^ TH Research Grade #5401135001 in 10 ml HBSS), and gently stirred at room temperature for 1 minute and then incubated in a 37 °C water bath for 5 min. The digest was collected in 20 ml high glucose DMEM with 10% heat-inactivated Fetal Bovine Serum (FBS). The digestion process was repeated six times. After passing through a 75 μm mesh steel filter 6–8 times, the filtrate was collected and centrifuged at 1200 rpm for 5 min and re-suspended in serum-containing media (10% FBS, 1% penicillin-streptomycin, high glucose DMEM). The cells were pre-plated in 100 mm TC-Treated Culture Dish (Corning, NY, USA) for 1.5 hours to attach non-cardiac cells. After centrifugation and re-sustentation in the medium (10% FBS, 1% penicillin-streptomycin, 0.1 mM BrdU, low glucose DMEM), the density of non-attached cells was counted using a haemocytometer and plated into 6-well culture plates or 60mm dishes. After 24 hours, the culture medium was replaced with new low glucose DMEM containing 10% FBS.

### Construction of lentiviral expression vector containing LAMIN A/C gene

Within the template of Lamin A/C gene, a specific fragment was amplified by PCR. The primers were as follows:

Lamin A/C c.646-MF, AGTGAGGAGCTGTGTGAGACCAAGCGCCGTC;

Lamin A/C c.646-MR, GCTTGGTCTCACACAGCTCCTCACTGTAGAT;

Lamin A/C c.1135-MF, GCCTACCGCAAGGTCTTGGAGGGCGAGGAGG;

Lamin A/C c.1135-MR, CGCCCTCCAAGACCTTGCGGTAGGCGTGGAT.

HEK293T cells were seeded into 10-cm culture dish. Transfection were performed with Lipofectamine2000 (Invitrogen) when cells were 90% confluent. Briefly, each dish were transfected with 5 μg lentivirus vectors containing the Lamin A/C cDNA (NM_170707.3), 3 μg helper plasmid containing Gag/Pol/Rev (Helper 1) and 2 μg helper plasmid containing the VSVG envelope. Supernatant were collected 24 hours and 48 hours post transfection. Then the virus were concentrated by ultracentrifugation (Beckman optimaL-90K, type 50.2 rotor, 50000 rpm for 4 hours).

### Western blot

Cells were lysed using radioimmunoprecipitation assay (RIPA) buffer with freshly added protease inhibitor cocktail (Roche, Mannheim, Germany). The cells were scraped off using a cell scraper and incubated on ice for 30 minutes. The lysate was then cleared by centrifuging at 13,000 g at 4 °C. Protein concentration was estimated by Enhanced BCA Protein Assay Kit (Beyotime, Beijing, China). 30 μg of protein resuspended in Laemmli sample buffer was loaded per sample. Denatured proteins were resolved on 4–12% Nu-PAGE bis-tris polyacrylamide gels and blotted to a polyvinylidene fluoride (PVDF) membrane. Blocking was done with incubation in 10% non-fat dry milk in tris-buffered saline (TBS) with 0.1% Tween-20. The membrane was then probed with primary antibodies in 5% milk in TBST at 4 °C overnight and sequentially detected with horseradish peroxidase conjugated secondary antibodies. The signal was revealed by autoradiography using enhanced chemiluminescence (ECL) (Pierce, Thermo Fisher Scientific Inc. Hampshire, UK).

### Terminal deoxynucleotidyl transferase dUTP nick end labeling (TUNEL) assay

TUNEL assay was performed using *In Situ* Cell Death Detection Kit, Fluorescein (Roche Applied Sciences, Mannheim, Germany) according to the manufacturer’s protocol. Cells were grown on glass coverslips, and after assigned treatment, cells were fixed and permeabilized with 0.1% Triton X-100 in 0.1% sodium citrate for 2 minutes on ice. Cells were then incubated in TUNEL reaction mixture at 37 °C for 60 minutes in a humidified dark chamber. Coverslips were mounted onto glycerol-based mountant. Images were collected and analyzed on a Zeiss LSM 700 confocal microscope (Carl Zeiss Inc., Germany).

### Immunofluorescence staining

Cells grown at subconfluency were collected and fixed with 4% paraformaldehyde/phosphate-buffered saline (PBS) and permeabilized with 0.2% Triton X-100 in phosphate-buffered saline for 10 minutes at room temperature. Cells grown at similar subconfluency for all cell lines tested. Primary antibodies in 5% bovine serum albumin in PBS with 0.05% Tween 20 were incubated for 1 hour at room temperature or overnight at 4 °C. Cells were washed three times and incubated with appropriate secondary antibodies for 1 hour at room temperature. Slides were mounted in Anti-Fade medium with 4,6- diamidino-2-phenylindole (DAPI) (Zhongshanjinqiao, Beijing, China). Images were collected and analyzed on a Zeiss LSM 700 confocal microscope (Carl Zeiss Inc., Germany). Images were captured using identical exposure times for each cell line.

## Statistical Analysis

Data are presented as mean ± SD. Significance was compared using SPSS statistical software (version 19.0, SPSS Inc.). Differences between mean values were determined using unpaired or paired Student t-test and ANOVA for multiple comparisons followed by the Bonferroni post-hoc test.

### Data availability

The datasets generated during and analyzed during the current study are available from the corresponding author on reasonable request.

## Electronic supplementary material

Supplementary information
